# Clinical effects of soluble interleukin-6 receptor detection on autoimmune rheumatic disease patients

**DOI:** 10.12669/pjms.304.5137

**Published:** 2014

**Authors:** Meng Chen, Ming-Hua Xu, Cai-Xia Sun, Zheng Zhang, Yong Du

**Affiliations:** 1 Meng Chen, Department of Rheumatology, Affiliated Hospital of Hebei University, Baoding 071000, P. R. China.; 2 Ming-Hua Xu, Department of Rheumatology, Affiliated Hospital of Hebei University, Baoding 071000, P. R. China.; 3 Cai-Xia Sun, Department of Rheumatology, Affiliated Hospital of Hebei University, Baoding 071000, P. R. China.; 4 Zheng Zhang, Department of Rheumatology, Affiliated Hospital of Hebei University, Baoding 071000, P. R. China.; 5 Yong Du, Department of Rheumatology, Affiliated Hospital of Hebei University, Baoding 071000, P. R. China.

**Keywords:** Autoimmune rheumatic disease, Detection, Diagnosis and treatment, Soluble interleukin-6 receptor

## Abstract

***Objective*** ***:*** This study aims to explore the effects of soluble interleukin-6 receptor (IL-6R) on the clinical diagnosis and treatment of autoimmune rheumatic disease (ARD) patients.

***Methods: ***Sixty-eight ARD patients enrolled in our hospital recently were selected, the IL-6R levels of whom were detected by double-antibody sandwich ELISA method and compared with the IL-6 levels of normal subjects. The expressions of IL-6R in joint tissues were detected by section staining. The mechanism regarding the effects of IL-6R was postulated by administering the patients with blocking agent.

***Results:*** The blood IL-6R level of ARD patients was 2-3 times higher than the IL-6 level of normal subjects with significant difference. The detection results of C-reactive protein and erythrocyte sedimentation rate show that both the indicators were significantly decreased (P=0.0098 and 0.0097 respectively). IL-6R was associated with autoimmunity based on the considerable expression in tissue sections, which was also verified by the alleviated symptoms after blocking IL-6R expression.

***Conclusion:*** Detecting soluble IL-6R is able to determine the patient's conditions and treatment effects. Meanwhile, soluble IL-6R detection can also serve as the inflammatory responses of ARD, as well as the determination index for abnormal immune responses and generated antibodies number, which is crucial for early diagnosis.

## INTRODUCTION

Autoimmune rheumatic disease (ARD) is often attributed to the attack of immune system by dysimmunity to autologous tissues. There are numerous pathogeneses for ARD, of which the destruction of cross immunity is most important.^1^ Immune system may kill autologous organisms by mistake because some bacteria and pathogens share the same heterophil antigens with human. Interleukin-6 (IL-6) is an important cytokine of T- and B-lymphocytes, the level of which indicates the status of immune function.^2^^,^^3^ Thus, the levels of IL-6 and its soluble receptor (IL-6R) in human body can often be used as indices for the diagnosis and detection of autoimmune diseases.

Our objective was to explore the effects of soluble interleukin-6 receptor (IL-6R) on the clinical diagnosis and treatment of autoimmune rheumatic disease (ARD) patients

## METHODS

***General*** ***I*** ***nformation: ***Sixty-eight ARD patients who had been treated in our hospital from 2010 to 2012 were selected in this study, including 39 males and 29 females. There were 16 cases of ankylosing spondylitis (AS), 23 cases of rheumatoid arthritis (RA), 19 cases of systemic lupus erythematosus (SLE) and 10 cases of polymyositis (PM). The IL-6R levels were analyzed and detected by double-antibody sandwich ELISA method and compared with the IL-6 levels of normal subjects. Meanwhile, the microscopic results of leukocytes in the patients and normal subjects under the intervention of IL-6 were compared.

***Detection*** ***M*** ***ethods: ***The levels of IL-6R were detected by double-antibody sandwich ELISA method. All apparatus and drugs were purchased from Immunotch Medical Products Co., Ltd. During the test, plasma sample and 150 μL of antibodies at different concentrations were added first to wells with alkaline phosphatase as the marker. After the addition of substrate (200 μL) to observe the reaction of CH-segment enzyme as well as staining, the absorbance was calculated at the wavelength of 405 nm to obtain the measurement value of IL-6R.^4^

***Immunohistochemical*** ***S*** ***taining: ***Pathological and healthy joint tissues were collected from ARD patients and normal subjects for section preparation, to which were then dropwise added known labeled antibody. The sections were observed by light microscopy after being rinsed, based on which results were obtained.

***Treatment*** ***M*** ***ethods: ***Blocking agent was used to study the correlative factors of IL-6R in autoimmunity. The patients were administered according to weights once (8 mg/kg) every four weeks. After eight weeks of treatment, C-reactive protein levels and erythrocyte sedimentation rates were detected to observe therapeutic effects.^5^^-^^7^

***Statistical*** ***A*** ***nalysis: ***All data were analyzed by SPSS 15.0. The numerical data were expressed as (mean ± SD) and compared by paired t test. The correlations were calculated based on the experimental data of two groups by using the software package of SPSS15.0. P<0.05 was considered statistically significant.

## RESULTS

According to the results of double-antibody sandwich ELISA, the blood IL-6R level of ARD patients was 2-3 times higher than that of normal subjects, between which the difference was statistically significant. The results are listed in Table-I.

**Fig.1:**
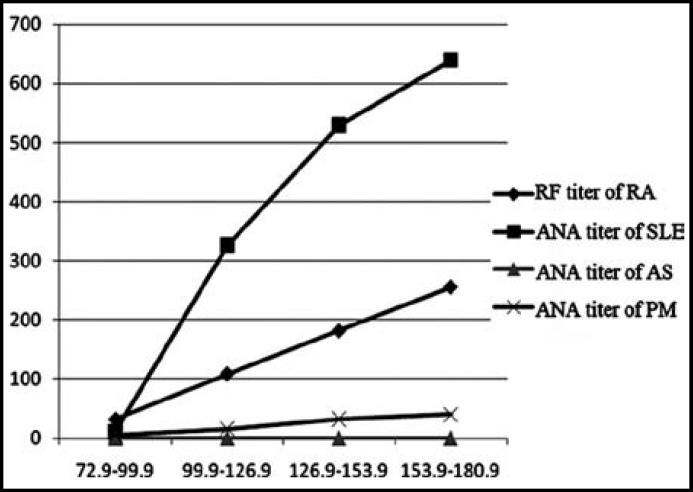
Correlation between soluble IL-6 level and titer

Meanwhile, the correlations between soluble IL-6R levels and titers of ANA, DNA, ENA and RF were studied, revealing that IL-6R levels in the patients were positively correlated with titers (Fig.1).

**Fig.2:**
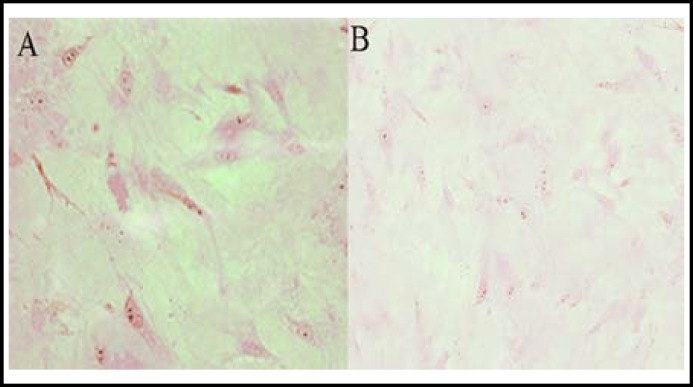
Anti-IL-6 staining sections of RA. A: Staining section of patient; B: Staining section of normal subject

**Fig.3:**
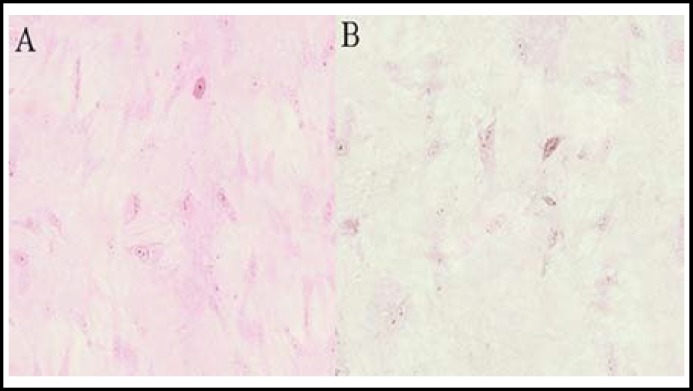
Anti-IL-6R staining sections of RA. A: Staining section of patient; B: Staining section of normal subject

Immunohistochemical staining method was used for tissue section staining and microscopic observation. We found that IL-6 and IL-6R existed in cells around the inflammatory tissues of RA patients, inferring that IL-6 was correlated with ARD joints, and played an essential role in its pathogenesis, the effect of which is evident in allergic reaction-related parts (Fig. 2 and 3).

At the same time, IL-6R blocker therapy was adopted for patients. The detection results of C-reactive protein and erythrocyte sedimentation rate show that both the indicators were somewhat decreased. The outcomes after treatment were significantly different from those before treatment (Table-II).

## DISCUSSION

IL-6, which is a key substance that mediates inflammatory response in human body, is mainly manifested in regulating physiological activities of various immune cells and controlling immune response. Soluble IL-6R, which is a glycoprotein attached to cell surface, can cooperate with IL-6 in regulating the proliferation and differentiation of immune cells, being more effective in regulating the physiological activities of T- and B-lymphocytes.^8^^-^^10^

Glycoprotein attached to the membrane of IL-sensitive cells may be prone to falling off in metabolism, thus forming soluble IL-6R after changes for a period of time. This receptor consists of ligand and non-ligand binding receptor protein chains, the molecular weights of which are 80 kD and 130 kD respectively. In human body, the complex of soluble IL-6R and IL-6 forms relying on the mediation of ligand binding receptor protein (80 kD), after which the complex binds non-ligand binding receptor protein to activate the binding of cells to IL-6, thus becoming IL-6-sensitive cells.^11^^-^^13^ In this study, the bloods of ARD patients and normal subjects were collected, which were reacted with same amounts of soluble IL-6 antibody detecting agents for comparison.

**Table-I:** IL-6 and IL-6R results of ARD patients and normal subjects

***Item***	***n***	***IL-6***	***IL-6R***
Autoimmune rheumatic disease	68	46.8±12.9	128.6±54.1
Normal	68	9.8±7.2	45.3±16.9
t value		20.65	12.11
r value		0.945	0.912
*P*		0.0054	0.0068

**Table-II:** C-reactive protein levels and erythrocyte sedimentation rates before and after treatment

***Item***	***n***	***C-reactive protein*** *** level***	***Erythrocyte sedimentation rate***
Before	68	26.3±16.2	54.5±22.3
After	68	6.1±4.5	23.6±7.2
Paired t test		4.53	6.49
r value		0.763	0.782
*P value*		0.0098	0.0097

ARD patients had a higher proportion of soluble IL-6R than normal subjects, which was also positively correlated with the severity. It has previously been reported that some autoimmune diseases may be accompanied by an increase in IL-6 level probably related with the pathological changes in human body (e.g. tumorigenesis). The emergence of a large number of IL-6 may aggravate the inflammatory response in patients.^14^^-^^16^ The specific reason remains undefined, but it is estimated that the increase of soluble IL-6R can enhance the binding ability of non-ligand binding receptor protein involved in cytokine signal transduction to IL-6R, thus rendering the complex of soluble IL-6R and IL-6 to reach limit that engenders immune responses in human body. Hence, abnormal immune responses occur, the symptoms of which may also be aggravated with increasing immunogens.^17^^-^^20^ In other words, soluble IL-6R indicates the appearance of immune response, the changes of which have close relationship with disease and treatment progress, and the strength of immune response. Detecting the changes of IL- 6 and soluble IL-6R levels in patients can more directly reflect the disease conditions, which can be used in clinic to auxiliarily determine the intensity of inflammatory response and post-treatment recovery.

Obviously, the rise of soluble IL-6R level is closely associated with the appearance of "acute-phase protein" in inflammation, and soluble IL-6R can also affect the gene expression of albumin in liver and inhibit the production of albumin to be significantly lower than the normal level and to produce other adverse reactions in human body. Soluble IL-6R regulates the interaction between IL-6 and cells, while in turn, IL-6 controls the expression level of soluble IL-6R. Moreover, the interaction synergetically influences the physiological activities of immune cells and abnormal immune response, which can thus be applied in clinic to monitor the adverse reactions in patients as an auxiliary treatment protocol.

## Authors’ Contributions:

**CM:** Manuscript preparation and study design.

**XMH, SCX, ZZ and DY:** Data collection and analysis.
